# Nursing Students’ Volunteer Experiences of Interacting with Children Receiving Pediatric Palliative Care: A Qualitative Study

**DOI:** 10.3390/children11111391

**Published:** 2024-11-16

**Authors:** Eunju Jin, Hyunju Kang

**Affiliations:** 1 Department of Nursing, Gangneung Yeongdong University, Gangneung 25521, Gangwon-do, Republic of Korea; 201915076@kangwon.ac.kr; 2 College of Nursing, Kangwon National University, Chuncheon 24341, Gangwon-do, Republic of Korea

**Keywords:** palliative care, pediatric nursing, qualitative research, students, nursing, volunteer

## Abstract

Background/Objectives: Pediatric palliative care refers to active, holistic care that provides support not only for families but also for the physical, psychological, social, and spiritual needs of pediatric patients with severe life-threatening diseases. Nursing students’ volunteer work for pediatric patients requiring palliative care is a unique and special experience with which to understand them as prospective medical personnel and that allows them to directly experience and feel the needs and reality related to emotional support. This study aimed to explore the experiences of nursing students who volunteer in pediatric in palliative care settings. Methods: The participants, selected through purposive sampling, were 20 nursing undergraduate students who volunteered at a pediatric palliative care medical center or a private community organization providing a support program for pediatric patients receiving palliative care. Individual in-depth interviews were conducted, and data were analyzed using content analysis. Data were collected from 7 August to 27 November 2023. Results: Five major categories were derived: (1) meeting with children—the process of facing and overcoming challenges; (2) the journey of changing through interactions with children; (3) parting with the child—anticipation, shock, and remembering; (4) new insights into pediatric palliative care; (5) and growing as a nursing student. Conclusions: The volunteer activities enabled the nursing students to interact with pediatric patients undergoing palliative care outside the sphere of clinical education. It increased these students’ awareness of palliative care and provided an opportunity for self-reflection and growth. It also provided an opportunity to improve empathy and provide emotional support.

## 1. Introduction

With advances in medical treatment, the survival rate for patients with life-threatening diseases has significantly increased [1]. Accordingly, the number of children and adolescents living with complex or severe chronic diseases and disabilities with uncertain prognoses has also increased [1]. Pediatric palliative care refers to active, holistic care that provides support not only for families but also for the physical, psychological, social, and spiritual needs of pediatric patients with life-threatening diseases [2]. The World Health Organization views palliative care as a key factor within the healthcare system and emphasizes the importance of adopting a universal approach to ensure that all patients in need of palliative care receive high-quality services without restrictions [3].

As part of these efforts, South Korea launched a pilot project for pediatric palliative care in 2018. As of 2023, 11 hospitals nationwide were expanding this project [1]. The purpose of this pilot project is to provide not only palliative care services at tertiary hospitals, community medical institutions, and homes through a multidisciplinary team but also specialized education on palliative care for children and adolescents [1]. A multidisciplinary palliative care team may include pain specialists, pharmacists, physical therapists, nutritionists, and volunteers, in addition to doctors, nurses, social workers, psychotherapists, and spiritual care providers, to meet the needs of patients and caregivers and improve their quality of life [3,4]. Hospice palliative care volunteers provide comfort and support to patients and their families through patient visits, physical care, religious or leisure activities, and bereavement family care [5]. With the recent expansion of domestic pediatric palliative care projects, opportunities for volunteers to participate in pediatric care have also increased in number. In pediatric palliative care, volunteers play an important supportive role within the palliative care team by spending time with pediatric patients and their families, allowing them to express the anxiety and depression they experience, or sharing administrative tasks accompanied by age-appropriate treatment, games, or activities [5,6].

In previous studies, it was found that not only middle-aged adults but also college students and young adults participated as hospice palliative care volunteers [5,6,7]. Undergraduate volunteering provides opportunities to improve social responsibility, knowledge, and life skills. For nursing students, volunteer activities related to their majors present an opportunity to not only apply the nursing knowledge acquired, evaluate their abilities, and explore their majors but also establish the role and identity of the nursing profession within the community [8]. Previous studies have closely examined the experiences and perceptions of domestic nursing students through local volunteering related to their majors, focusing on volunteer experiences for the elderly with dementia in the community and volunteer activities at local nursing institutions [9,10]. In China, nursing students were recognized as having helped terminally ill patients through volunteer work, endowing them with the ability and confidence required to care for the patients and themselves [11]. In particular, volunteering in community and palliative care settings enhances the empathy of undergraduate nursing and medical students [12].

In South Korea, pediatric palliative care volunteers, including nursing students, participate in hospital-based pediatric palliative care projects. Volunteers engage with children with complex or severe chronic diseases who are either hospitalized for treatment or awaiting outpatient care. There are also volunteer programs that allow volunteers to meet children in need of palliative care within the community, where they can regularly interact with the children and support them. Notably, children requiring palliative care have special care needs, and additional knowledge and skills such as family-centered care and age-appropriate communication are essential [13]. In fact, the pediatric patients nursing students meet through volunteer work, especially those receiving palliative care, are often not easily accessible in clinical practice. Additionally, the concept of pediatric palliative care is abstract and complex for nursing students. This can create a learning dilemma due to the gap between theory and practice in the nursing curriculum. Therefore, nursing students’ participation in volunteering for pediatric patients receiving palliative care is a unique and special experience that allows students to understand and empathize with the patients and directly experience and feel the needs and reality of care as prospective medical personnel. Thus, comprehending its significance through qualitative research is imperative.

Therefore, this study aimed to explore and understand nursing students’ experiences of volunteering for pediatric patients receiving palliative care through qualitative research, addressing the following question: “What is the experience of nursing students volunteering for pediatric patients receiving palliative care?” As the role of nursing student volunteers does not entail being directly involved in palliative care but interacting with children, the focus should be on understanding their experiences and intrinsic changes through these interactions. It provides evidence that can be sued to establish educational directions and strategies for improving nursing students’ pediatric palliative care competencies against the background of the expanding pediatric palliative service in South Korea.

## 2. Methods

The standard of rigor in this study was based on Sandelowski’s criteria for qualitative research [14], and this study followed the Consolidated Criteria for Reporting Qualitative Research (COREQ) reporting guidelines [15].

### 2.1. Participants and Settings

The participants were selected through purposive sampling. First, the researchers obtained consent from two institutions in South Korea. One was the pediatric palliative care project group of a tertiary hospital, and the other was a private community foundation, which grants the wishes of children battling critical illnesses such as cancer. Volunteering in the hospital involved one-on-one interactions with children who were either hospitalized or waiting in outpatient settings, engaging in the play activities of the child’s choosing. Volunteering for the private community organization involved working in teams to carry out a step-by-step project aimed at fulfilling a child’s wish. Next, recruitment notices were posted on the online community sites used by the university student volunteers from these two institutions, through which participants were recruited. The criteria for selection were as follows: participants had to be aged 18 years or older, have directly participated in volunteering programs for pediatric patients receiving palliative care, have had interaction experiences with children, have expressed a willingness to actively share those experiences through interviews, and either have been enrolled in a nursing school at the time of the study or nursing graduates who were nursing students at the time of the volunteer activities. The researchers explained the details of the study verbally to those who were willing to participate, who were then selected as participants. None of the participants withdrew from the interview partway through.

### 2.2. Data Collection

Data were collected from 7 August to 27 November 2023 through one-to-one interviews. Two researchers collected data through in-depth individual interviews with participants to help the research team understand the more complex experiences through various interpretations by multiple researchers. Both researchers had PhDs and a specialty of pediatric nursing, were professors at a nursing school, had undergone training in qualitative research methods, and were experienced in conducting qualitative research through individual interviews, and one of them is a pediatric nurse practitioner. The interviews were not conducted between professors and students at the same university to allow the participants to engage in the interviews freely. The interviews were conducted at a quiet café or school at a time convenient for the participants so that they could talk freely. If participants preferred not to have face-to-face interviews, a real-time online video conference system (Zoom) was used instead, for which the researchers provided the access link in advance. Zoom recording or Samsung mobile recording software (v21.4.00.34) was used.

At the beginning of each interview, the corresponding researcher and participant briefly greeted each other, creating a comfortable atmosphere, and the researcher briefly explained the purpose and topic of this study. Subsequently, the interview was conducted using semi-structured questions, tailored in a way that allowed participants to express their experiences as broadly and deeply as possible. Participants were informed in advance that all interviews would be audio-recorded. The researchers observed the participants’ nonverbal behaviors and the interview atmosphere on the day of the interview, took notes, and referred to them for analysis.

Interviews were conducted once for each participant, lasting 50 to 90 min. After the interview, the researcher directly transcribed the recorded audio file. During analysis, data collection was terminated when it was determined that the same content was repeated, no new content was released, and data saturation was reached. The total sample comprised 20 participants, none of whom refused to be interviewed or withdrew from the study. The main questions guiding the interview were “How did you start volunteering for pediatric patients receiving palliative care as a nursing student?”, “What was the hard part about volunteering?”, “What did you gain from volunteering?”, “What is the role of a nursing student in volunteering?”, “How did your experience affect your major in nursing?”, and “What did you feel is necessary to volunteer for pediatric patients receiving palliative care?”

### 2.3. Data Analysis

Data analysis was conducted by two researchers using the content analysis method developed by Hsieh and Shannon [16]. Content analysis is a qualitative research method commonly used in the healthcare field to understand research phenomena and grasp content and contextual meaning by efficiently and systematically classifying large quantities of qualitative data collected through interviews [16].

First, the researchers repeatedly read the transcribed data and selected important sentences and paragraphs. Second, similar meanings were grouped and coded. Third, the derived meanings were collected to identify subcategories, which were checked to derive the main categories by grouping similar ones together. The researchers repeatedly discussed the results of the analysis until they reached a consensus. In addition, for the systematic analysis and management of data, MAXQDA 24, a qualitative data analysis software, was used to check the coding and category patterns of the entire dataset. Two participants provided feedback on the final results to ensure that they matched their statements.

### 2.4. Rigor

First, to ensure credibility, the researchers conducted interviews with an open attitude, recording all interview content. The transcribed data were analyzed using a content analysis manual, and the results were verified by two participants. Second, for fittingness, participants who could express their experiences in a rich and deep manner were selected until the data were saturated. Third, for auditability, the data collection and analysis processes were described in detail so that they could be traced, and a consensus of the results was reached through discussion between the researchers. Fourth, for confirmability, when presenting the analysis results, the participants’ views were quoted so that the reader could check them [13].

### 2.5. Ethical Considerations

This study was approved by the university’s institutional review board (KWNUIRB-2023-06-005-001). Prior to the interviews, the participants were informed of the purpose and process of this study, told that the interviews would be recorded, and given information regarding confidentiality. They were also informed that they had the right to withdraw from the study at any time. Interviews were conducted after obtaining consent from the participants. Their names were replaced with numbers in the study files to protect their privacy. After the interviews, participants received a gift as an expression of gratitude.

## 3. Results

Twenty participants were interviewed for this study. Their demographic characteristics are presented in Table 1. Most of the participants were in the third year or higher and did not follow a religion. All the participants in their third year or higher completed a pediatric nursing practicum. Most of the participants selected nursing because of their aptitudes and interests. Sixteen participants participated in the community foundation’s volunteer program, while the other four participated in the hospital palliative care team’s volunteer program. They met children, on average, 19.3 times through volunteering. Each student was assigned a child with palliative care needs during each visit, and the participant with the most frequent involvement had been volunteering for approximately four years.

From the collected data, 360 meaningful sentences and paragraphs were selected. This content was further divided into 16 subcategories and summarized into 5 categories. Table 2 presents the categories and subcategories.

***Category 1. Meeting with children: a process of facing and overcoming challenges***

This theme refers to the process in which the participants experience the initial shock of encountering severe illnesses, overcome communication difficulties, and feel a sense of responsibility while fulfilling a child’s wishes, ultimately helping them overcome these challenges.

**Subcategory 1-1. Witnessing severe illnesses and monitoring a child’s condition**

The participants encountered children in need of palliative care, such as those with childhood cancer and rare and incurable diseases, who they had not met before while volunteering. The ages and diseases of the pediatric patients varied. When the participants met the children, they had to engage in activities while taking into consideration each child’s condition.

*“I think I observed the ventilator closely for any emergencies and made sure that the tubes did not get tangled when moving the child with the help of the parents and that the ventilator tube was taut, while also checking for any changes in volume or mode. I also helped with ambulation and monitored the child’s condition.”*

*(Participant 13)*

*“The child had muscular dystrophy? A condition that causes muscle stiffening, so he was in a wheelchair and could only play games a little with his hands because he could not move freely.“*

*(Participant 9)*

**Subcategory 1-2. Overcoming communication difficulties with a child**

The participants experienced practical difficulties, as the volunteer activities were not as easy as expected. The participants became aware of the difficulty of the activities they were supposed to engage in when communication was not smooth due to severe diseases or a child’s age and adjusted them by changing the planned activities.

*“He was a lower-grade elementary school child who had cognitive impairment due to meningitis, and he had difficulty communicating, so we addressed his wishes offline and online by focusing more on hands-on activities or drawing instead of verbal communication…”*

*(Participant 17)*

**Subcategory 1-3. Struggling with the pressure of meeting children’s expectations**

They engaged in volunteer activities despite the burden of not being able to fulfil the child’s needs or wishes, always keeping the child’s condition in mind during their interactions.

*“I felt a lot of pressure thinking, ‘What if I cannot really help address their wishes or things fall below expectations?’… I was always nervous, and I worried a lot about whether I could meet their needs because I did not know when an emergency would occur.” (Participant 13)*

***Category 2. A journey of change through interactions with children***

This theme refers to the process in which the participants and children shared emotional connections, built trust through promises and anticipation, and experienced mutual positive impacts, leading to personal growth and change. The participants engaged in in-depth interactions while also continuing to meet with the children several times. In the process, they created meaningful relationships, became playmates who had fun with young children, and became friends and counsellors who could share their thoughts with older children. Since the impact of an illness varies depending on the developmental stage of the child, the participants found that the child’s age should be taken into consideration in interactions between pediatric palliative care volunteers and children.

**Subcategory 2-1. Becoming friends by sharing inner struggles**

The participants continuously met with adolescents, and, when they formed close bonds, they became friends and counselors with whom adolescents could share their concerns about school life or careers. The participants became friends who shared their pediatric patients’ hopes and wishes and waited with them for their next appointments, showing their solidarity with the patients.

*“I have become very close with the child, and she started confiding in me with her worries or things that are troubling for her these days or even things about her friends at school.”*

*(Participant 18)*

**Subcategory 2-2. Making promises and waiting together**

The participants met repeatedly, sharing their hopes and wishes, such as what they wanted to do with children and what they wanted to achieve, making the next appointment, and waiting for the next meeting. These meetings lifted the spirits of both the participants and the children.

*“I always ask what they like or what they want to do next. They say they want to make something next time…Then the children would be a little motivated to be a little bit stronger because we are going to do this together next week…”*

*(Participant 18)*

**Subcategory 2-3. Witnessing positive changes in children as they look forward to future meeting**

At first, the children began their activities according to the recommendations of hospitals and parents, but the participants observed changes in children who waited for meetings and pursued the guidelines of volunteer programs.

*“At first, he was really flat and blunt in his responses, so I did not know what to do. But over time, the parents told me that the child enjoys meeting us and is curious about when we will meet next.”*

*(Participant 11)*

***Category 3. Parting with the child: anticipation, shock, and remembering***

This theme was derived from the experiences of some of the participants. It represents the process in which the participants anticipated a child’s passing, prepared for their final meeting, coped with the sadness and shock after learning of the child’s death, and reflected on their memories with the child as they process their emotions. The participants sometimes encountered the deaths of their pediatric patients. It was difficult to keep in mind that any meetings with these children could be their last, and they were shocked by and afraid of hearing the news of a child’s death, simultaneously feeling sad for their family. After the death of a child, they collected their emotions as they went through the process of commemorating the child.

**Subcategory 3-1. Anticipating the final meeting**

Some participants sometimes struggled with knowing that a child’s condition could deteriorate and that a given meeting could be the last one.

*“It was kind of tough knowing that this activity might be the last thing for the child in their life…”*

*(Participant 5)*

*“The child was at risk of getting worse, with the cancer reaching her abdomen… I wrote that thinking that I would not see her again.”*

*(Participant 4)*

**Subcategory 3-2. Shock and grief** **expressed in the manner of** **a family member**

The participants were shocked when they heard that a child had actually died. They also felt profound sadness—as they would feel upon losing a family member—when they heard the news of a child’s death

*“When the organization sent a text saying the child has ‘gone on a journey,’ it hit me harder than I would expect.”*

*(Participant 1)*

*“Finding out the child has passed away hits you like a ton of bricks, almost like you are family.”*

*(Participant 3)*

***Subcategory 3-3. Reflecting on memories and managing emotions***

The participants recovered from their sadness and paid tribute to the children by recalling their memories of the children while also recalling the message of remembrance received from the hospital or the images they kept of the children. They felt this process was helpful.

*“Looking back at the child’s life, their name, what they liked, it helped me remember and honor them.”*

*(Participant 1)*

***Category 4. New insights into pediatric palliative care***

This theme represents the process in which the participants realize that children with severe illnesses continue to grow each day, discover the importance of sharing hope and joy, and recognize the value of family support, ultimately leading to a shift in their perspective on pediatric palliative care. They met children who were similar to other healthy children, smiling and exuding happiness, causing the participants to reflect on and change their preconceived notions about children sick with severe disease. The participants expanded their perspectives on pediatric palliative care by volunteering. Although the children were sick patients who needed palliative care, the participants believed in extending support to the parents and families of the children during the difficult treatment process. They also realized that volunteering in pediatric palliative care is a valuable activity, offering opportunities to find joy and share hope with children.

**Subcategory 4-1. Children who are ill but growing every day**

The participants learned that the children were struggling with pain but were still growing. The appearances of the children updated the participants’ existing perspectives on the subject of pediatric palliative care.

*“Children are still children, right? Even when they are sick, they grow. Every time I saw them, they seemed a bit taller.”*

*(Participant 1)*

**Subcategory 4-2. Importance of sharing joy and hope together**

The participants realized that pediatric palliative care volunteering is a valuable activity that brings them pleasure and renews hope in children, thereby empowering them.

*“I think that the child must have had so many tough moments wondering why they were different, but doing the activities with us and just laughing and having fun and focusing on the wish and all the good stuff seemed to give them the strength to keep going.”*

*(Participant 17)*

**Subcategory 4-3. Discovering the value of family support**

They also witnessed the difficulties of the families of the children, and they were able to understand their feelings and believed that support for them was necessary.

*“Seeing the mom cry despite everything, you just get how much she has been holding on. It was a real eye-opener for me, understanding how caregivers feel, something I could not have grasped before being in their shoes.”*

*(Participant 15)*

***Category 5. Growing as a nursing student***

This theme refers to the experience in which nursing students, through the joy, fulfilment, and confidence gained from volunteering in pediatric palliative care, begin to find their role as nursing students and develop motivation for their careers and nursing studies. The participants also had the opportunity to reflect on themselves and grow through volunteer activities. They felt joy and fulfillment in looking after children, set expectations for them, and were confident in meeting them in the future. The participants had an experience that prompted them to contemplate nursing through volunteer activities. In meetings with the children, they searched for opportunities to provide care as nursing students and actually realized the importance of emotional support in nursing. It was also an opportunity to find the will and motivation for studying while pondering the career path related to pediatric palliative care.

**Subcategory 5-1. Finding fulfilment and gaining confidence through meeting children**

The participants experienced joy and fulfilment induced by children who liked, followed, and greatly valued them. The participants gained confidence and set expectations for dealing with the children while continuing to meet them.

*“I feel like meeting this child was like getting a gift I would have never gotten otherwise… It is something priceless, and that is what means the most to me.”*

*(Participant 3)*

*“In the past, I used to be kind of scared in the pediatric ICU or around little children, but now I am feeling a bit more confident. Like, I can do this, and having this experience…I feel like my mind has opened up a bit.”*

*(Participant 19)*

**Subcategory 5-2. Investigating the role of a nursing student**

The participants volunteered for and identified different roles while trying to understand and examine each child’s condition as a nursing student. If they could do anything to help with the children’s treatment, they were willing to help.

*“We kept an eye on the child’s health, thinking about their condition. It was important to us that the child was having fun but even more important that they were not getting too tired.”*

*(Participant 3)*

*“Since the child had an illness, being a nursing student made me think I had to be extra careful about certain things because of their condition.”*

*(Participant 17)*

**Subcategory 5-3. Realizing the importance of emotional support**

The participants directly realized the value of emotional support in palliative care volunteering.

*“I think what we can do is give them emotional support, and that is one aspect of palliative care we can offer.”*

*(Participant 16)*

**Subcategory 5-4. Developing motivation for career and academic goals**

They also seriously considered career paths related to pediatric palliative care, thinking that it would be good to work in a palliative care ward. And they became interested in pediatric patients and were motivated to study nursing again.

*“Having this experience made me think that I could work in a pediatric palliative care unit in the future or take care of children on those floors. It really influenced my career choice.”*

*(Participant 1)*

*“After this, I got more interested in pediatric patients, and during my pediatric nursing course and clinical rotation, I found myself paying more attention to the children.”*

*(Participant 2)*

## 4. Discussion

This study was conducted to understand nursing students’ experiences of interacting with children receiving pediatric palliative care during their volunteer activities. In our study, the participants reported their experiences across 5 categories and 16 sub-categories.

First, the theme of “Meeting with children: a process of facing and overcoming challenges” was derived. The participants met children who had life-threatening diseases, such as cancer and congenital diseases, and needed pediatric palliative care. Because they had to meet children of various ages and with various diseases, they applied their knowledge of children’s development stages and assessed the health condition of the ill children. These activities became actual experiences rather than examples only encountered in nursing theory. Clinical nursing practice for nursing students in South Korea is very limited [17], and the experience of meeting children with severe disease through volunteering was a challenge that brought new levels of tension to the nursing students. Direct encounters with children in need of palliative care were also moments in which the students actually understood that these children were active and wanted to behave like other children of that age and not just lie in sick rooms. This practical knowledge challenged existing prejudices. In particular, they learned to communicate with pediatric patients with severe diseases. A previous study on a palliative care theory curriculum for nursing students reported a better understanding of patients and improved communication with patients [18]. Considering these results, the experience of pediatric palliative care volunteering seems to have been an opportunity for nursing students to build relationships with children and develop communication skills. Volunteering was difficult for the participants. In particular, they found the burdens of having to change their activities according to the condition of a sick child or to ensure that a child’s wishes were fulfilled to be difficult. This is comparable to the findings of a study on the burden of hospice volunteers, which reported that the burden of patient care was the highest and that volunteers felt guilty when they did not do their best for the patient [19]. In other words, a situation in which a child’s desire was not fulfilled was considered to be unsuccessful because the participants did not perform at their best level. On the other hand, since clinical nursing practice involves applying nursing knowledge, critically addressing patients’ health problems, and carrying out nursing interventions, the need to support students in gaining these experiences through clinical practice is further emphasized.

Second, the theme of “A journey of change through interactions with children” was derived. The participants continued meeting the children several times. During these meetings, they became joyful playmates for young children and the friends of older children who shared their worries and concerns with them. Through these interactions, the children looked forward to the volunteer work, and they experienced waiting for their next appointment together while sharing their hopes and wishes. In most clinical settings, nurses care for children by creating cooperative partnerships with their parents [20]. However, pediatric palliative care volunteers focused more on their relationships and interactions with the children rather than with their parents. They understood the children by forming relationships with them, in which their developmental characteristics were considered. In addition, sharing wishes with children conveys a message of hope while waiting for the next appointment and strengthens the value of interactions with pediatric patients. When the participants approached the children sincerely and carried out their activities with responsibility, the children became more open and expressed a desire to build a deeper relationship. This interaction enhanced the value of care provided through palliative care volunteering and became a meaningful part of the children’s lives.

Third, the theme of “Parting with the child: anticipation, shock, and remembering” was derived. Some participants were shocked, afraid, and saddened when they heard news of a child who had died or was near death. Through a support program for volunteers, the participants cherished the memories of a deceased child, helping them calm themselves. Medical staff caring for children receiving palliative care in hospitals with life-threatening conditions sometimes encounter the death of children but are not prepared for it or do not know how to handle it [13]. However, in pediatric palliative care, as a part of clinical nursing practice, because children and families are cared for in the context of children’s deaths, education is needed to help medical staff prepare children and families for understanding death and reflect on positive memories [21]. Nursing students who volunteer in pediatric palliative care are also indirectly placed in this situation; therefore, education and support regarding dealing with the deaths of children are needed. Since it is anticipated that some pediatric palliative care volunteer students may experience such things, there is a need to provide information on counseling centers or support programs within the university to ensure they receive sufficient counseling and support.

Fourth, the theme of “New insights into pediatric palliative care” was derived. While volunteering, the participants learned that a child in need of palliative care is sick but still growing; they watched struggling families during a child’s treatment process and realized that they needed to provide support for the family. They also realized that volunteering in pediatric palliative care is a valuable activity that brings them joy and the ability to share hope with children. The participants realized that even though children with severe illnesses require palliative care, they still want to have fun, make wishes, and pursue their dreams. Through this realization, the participants experienced a shift in their preconceived notions about children receiving palliative care. Pediatric palliative care refers to care that improves quality of life via using an interdisciplinary healthcare team approach to address, respect, and support the needs of children and families by providing developmentally appropriate individualized treatment and relief of holistic pain to children throughout the course of treatment of life-limiting diseases, regardless of the outcome [22]. While having to provide pediatric palliative care is very rare in the undergraduate curriculum [13], the volunteer experience of nursing students led to an understanding of the importance of pediatric palliative care and expanded perspectives.

Lastly, the theme of “Growing as a nursing student” was derived. The participants found joy and fulfilment through volunteer work and were confident in meeting sick children. Self-reflection is also a driving force behind the will to continue down the path of sacrifice and service. This is similar to findings from other studies in which participants experienced changes through volunteer work [23], realized the true joy and meaning of volunteer work, and achieved self-growth [24]. Since the satisfaction provoked by volunteering affects one’s willingness to continue volunteer activities [25], even if the pediatric palliative care volunteers began volunteering for various reasons, the reflection process compelled them to continue volunteering. The participants’ volunteer experiences transcended simple service and instead entailed identifying the roles of nursing students, realizing the importance of emotional support in nursing care, and motivating them to study nursing further. The participants recognized and viewed their experiences from the perspective of nursing and regarded them as areas that they should strive for and develop. In particular, they expected to perform related tasks while considering their future career paths. This is similar to the results of a study in which nursing students took pride in volunteer work and envisioned the future alongside their patients [10].

In South Korea, only a few hospitals continue to carry out pediatric palliative care projects, and student participation is limited. Support and organizational activities for pediatric palliative care are still insufficient in the community. However, there is growing interest in patients and families who need pediatric palliative care; accordingly, the development of various palliative care services by the government is necessary [1]. In addition to the education imparted by local institutions and hospitals, it is necessary to not only promote volunteering among the general public but also establish a regional pediatric palliative care service delivery system. Thus, expanding the opportunity to volunteer not only for nursing students, who are prospective medical personnel, but also for the general public will contribute to promoting pediatric palliative care and providing necessary care to children, constituting a major component of the multidisciplinary team approach. Such volunteer experiences help nursing students enhance their empathy and improve their ability to engage more deeply in interactions with patients [12]. Furthermore, expanding volunteer opportunities will raise awareness of pediatric palliative care and contribute to providing high-quality service.

This study has some limitations. First, there is a limit to the generalizability of the results to other institutions’ volunteer activities because this study only targeted nursing students who volunteered for pediatric palliative care at one university hospital and one local organization. Second, children in need of pediatric palliative care are unique and diverse; therefore, there is a limit to the degree to which all the relevant content can be encompassed by pediatric palliative care volunteer training. Third, in this study, interviews were not conducted between professors and students from the same university; however, there may be potential limitations or biases when a professor interviews students. Nevertheless, this study is meaningful because it explores and describes nursing students’ experiences of volunteer activities related to pediatric palliative care. Through this study, nursing students will be able to acquire new perspectives on pediatric palliative care through the lens of volunteer activities and make the most of opportunities for individual growth and reflection in relation to their majors, thereby expanding the opportunities for nursing students to participate in pediatric palliative care volunteer work. The results of this study indicate that providing nursing students with practical experience in interacting with children receiving palliative care, beyond traditional clinical training, can enhance their understanding of pediatric palliative care and improve their empathy. Such volunteer activities may contribute to preparing them to deliver nursing services in pediatric palliative care. We suggest expanding the opportunities for volunteer participation in hospitals and communities and conducting further research addressing the growth experiences of nursing students.

## 5. Conclusions

This qualitative study used content analysis to understand nursing students’ experiences of volunteering in a pediatric palliative care context. A total of five categories were derived. The participants met children in need of palliative care on various occasions, continued to meet them, interacted with them according to their developmental ages, and developed friendships with them. However, upon hearing the news of a child’s death, they felt shock and sadness and cherished the memory of the child. The volunteer activities involved practical difficulties, but they also presented an opportunity to experience joy, reap rewards, and pursue personal growth. Furthermore, the participants’ perspective on pediatric palliative care expanded, inspiring contemplation of academic pursuits and future career paths. Nursing students’ volunteer activities in pediatric palliative care raise awareness of palliative care and present an opportunity for self-reflection and growth. Various programs should be planned for nursing students in collaboration with hospitals and communities. Our study’s findings are meaningful in that they confirm and enhance the understanding of the meaning of nursing students’ experiences of volunteering for pediatric palliative care and are expected to be used to guide and direct pediatric palliative care education for nursing students in the future. Furthermore, the findings of this study are meaningful, as they could provide valuable insights for guiding the future direction of nursing education.

## Figures and Tables

**Table 1:** Demographic characteristics of the participants (n = 20).

Characteristics		Mean (SD, min–max) or n (%)
Gender	Female	20 (100)
Age (years)		22.5 (1.3, 20–27)
School year	Second year	2 (10)
	Third year	12 (60)
	Fourth year	2 (10)
	Graduate for the current year	4 (20)
Religion	Protestantism	6 (30)
	Catholism	1 (5)
	Buddhism	1 (5)
	None	12 (60)
Motivation for seeking admission into a nursing school	Aptitude and interest	14 (70)
	High employment rate	4 (20)
	Grades	2 (10)
Place of volunteering	Hospital	4 (20)
	Community	16 (80)
Number of meeting children met through volunteering		19.3 (16.3, 3–90)

**Table 2:** Categories and subcategories.

Categories	Subcategories
1. Meeting with children: a process of facing and overcoming challenges	1-1. Witnessing severe illnesses and monitoring the child’s condition 1-2. Overcoming communication difficulties with the child 1-3. Struggling with the pressure of meeting children’s expectations
2. A journey of change through interactions with children	2-1. Becoming friends by sharing inner struggles 2-2. Making promises and waiting together 2-3. Witnessing positive changes in children as they look forward to future meetings
3. Parting with the child: anticipation, shock, and remembering	3-1. Anticipating the final meeting 3-2. Shock and grief expressed in the manner of a family member 3-3. Reflecting on memories and managing emotions
4. New insights into pediatric palliative care	4-1. Children who are ill but growing every day 4-2. Importance of sharing joy and hope together 4-3. Discovering the value of family support
5. Growing as a nursing student	5-1. Gaining fulfilment and confidence through meeting children 5-2. Investigating the role of a nursing student 5-3. Realizing the importance of emotional support 5-4. Developing motivation for career and academic goals

## Data Availability

The data are not publicly available due to ethical reasons but are available on request from the corresponding author.
